# Transient receptor potential melastatin 8 (TRPM8) channels are involved in body temperature regulation

**DOI:** 10.1186/1744-8069-8-36

**Published:** 2012-05-09

**Authors:** Narender R Gavva, Carl Davis, Sonya G Lehto, Sara Rao, Weiya Wang, Dawn XD Zhu

**Affiliations:** 1 Department of Neuroscience, Amgen, One Amgen Center Drive, Thousand Oaks, CA, 91320, USA; 2 Department of Pharmacokinetics and Drug Metabolism, Amgen Inc, Thousand Oaks, CA, 91320-1799, USA

**Keywords:** TRPM8 antagonist, AMG0635, AMG2850, AMG8788, AMG9678, Compound 496, Body temperature regulation

## Abstract

**Background:**

Transient receptor potential cation channel subfamily M member 8 (TRPM8) is activated by cold temperature *in vitro* and has been demonstrated to act as a ‘cold temperature sensor’ *in vivo*. Although it is known that agonists of this ‘cold temperature sensor’, such as menthol and icilin, cause a transient increase in body temperature (T_b_), it is not known if TRPM8 plays a role in T_b_ regulation. Since TRPM8 has been considered as a potential target for chronic pain therapeutics, we have investigated the role of TRPM8 in T_b_ regulation.

**Results:**

We characterized five chemically distinct compounds (AMG0635, AMG2850, AMG8788, AMG9678, and Compound 496) as potent and selective antagonists of TRPM8 and tested their effects on T_b_ in rats and mice implanted with radiotelemetry probes. All five antagonists used in the study caused a transient decrease in T_b_ (maximum decrease of 0.98°C). Since thermoregulation is a homeostatic process that maintains T_b_ about 37°C, we further evaluated whether repeated administration of an antagonist attenuated the decrease in T_b_. Indeed, repeated daily administration of AMG9678 for four consecutive days showed a reduction in the magnitude of the T_b_ decrease Day 2 onwards.

**Conclusions:**

The data reported here demonstrate that TRPM8 channels play a role in T_b_ regulation. Further, a reduction of magnitude in T_b_ decrease after repeated dosing of an antagonist suggests that TRPM8’s role in T_b_ maintenance may not pose an issue for developing TRPM8 antagonists as therapeutics.

## Background

Cold sensation is derived from activation of the somatosensory system by a cold stimulus. Studies by Hansel and Zimmerman in the 1950s demonstrated that cold temperatures evoke action potentials in peripheral nerves [1,2]. Further, they have linked the effects of menthol to cold-responsive fibers by showing that menthol shifts the activation of cold-responsive fibers to warmer temperatures [3]. Calcium imaging and patch clamp studies in dissociated trigeminal and dorsal root ganglion neurons have revealed that cold stimuli induce calcium influx, suggesting direct opening of calcium-permeable ion channels by cold [4-7]. Search for an ion channel that responds to menthol and cold led to the cloning of TRPM8 that is activated by cold stimuli of <28.4°C [8,9]. TRPM8 is also activated by compounds that elicit a cooling sensation such as icilin (AG-3-5) [9] and its analogues, as well as endogenous lysophospholipids [10] and PIP_2_[11].

A number of TRP channels are activated at distinct ranges of temperature that span from noxious cold to noxious heat and are believed to act as thermosensors *in vivo*[12]*,* hence named ‘thermoTRPs’ [13]. Knockout mouse studies revealed that i) TRPV1 is required for hot temperature sensing [14], ii) TRPV3 is required for warm temperature sensing [15], iii) TRPV4 is required for warm temperature discrimination [16], and iv) TRPM8 is required for sensing innocuous ambient cold temperatures [17-21]. TRPA1 is reported to be activated by noxious cold (<10°C) *in vitro*[22,23], and to act as a noxious cold sensor *in vivo*[24]. Further, TRPA1 and TRPM8 have been reported to play a role in cold hypersensitivity [25,26]. Correlating with the cold sensing function, TRPM8 is expressed in the sensory neurons of the trigeminal and dorsal root ganglia and the peripheral nerve endings in the areas of the body that could be exposed to environmental cold temperatures (skin, oral cavity, inner ear, and nasal mucosa) [6,8,9,27-29].

TRP channel agonists such as capsaicin [30,31], resiniferatoxin [30], menthol [32], and icilin [33] are known to alter T_b_; however, the involvement of TRP channels in the regulation of T_b_ was not known definitively until recently (reviewed in [12,31]). We have reported that TRPV1 is tonically active *in vivo* and involved in T_b_ maintenance [34,35] by negative modulation of thermogenesis and vasoconstriction based on the fact that i) a variety of TRPV1 antagonists caused hyperthermia in multiple species [34], ii) TRPV1 antagonists did not cause hyperthermia in TRPV1 knockout mice [35], and iii) TRPV1 antagonists increase thermogenesis and vasoconstriction [35]. Further, clinical studies demonstrated that TRPV1 antagonists cause a rightward shift in heat tolerance by 2–4°C [36,37] suggesting the impairment of heat detection confirming the ‘heat sensor’ function of TRPV1. Menthol and icilin have been reported to cause a transient rise in T_b_[32,33,38,39], and it was also demonstrated that the menthol and cold temperature induced increase in T_b_ is TRPM8 mediated (i.e., both menthol and cold temperature caused an increase in T_b_ only in wild type but not in TRPM8 knockout mice) [40]. However, it is not known if TRPM8 itself is tonically active or even if it is involved in homeostatic maintenance of T_b_. Here, we report the characterization of novel TRPM8 antagonists and their effect on T_b_. Based on the data presented here we conclude that TRPM8 channels play a role in T_b_ regulation.

## Results

### Characterization of TRPM8 antagonists

**Figure 1:**
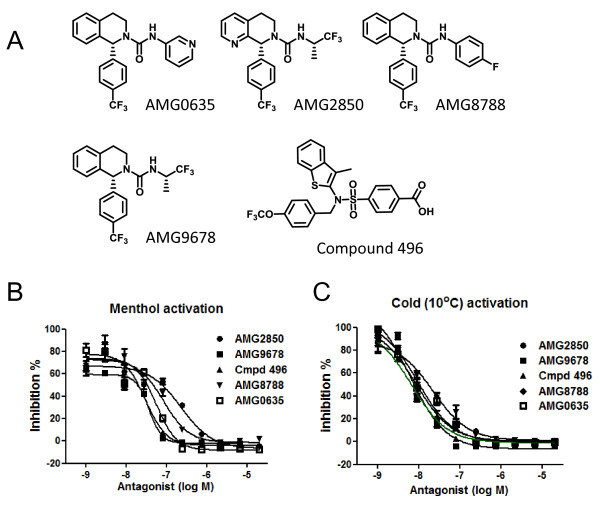
** Characterization of five distinct compounds as TRPM8 antagonists. A**) chemical structures of antagonists used in the study. **B**) Concentration dependent effects of antagonists on menthol-induced intracellular calcium increase in CHO cells stably expressing rat TRPM8. **C**) Concentration dependent effects of antagonists on cold (10°C)-induced intracellular calcium increase in CHO cells stably expressing rat TRPM8. Each data point in the graph are average ± S.D. of an experiment conducted in triplicate.

In our efforts to identify TRPM8 antagonists, we screened compound libraries and found several chemotypes that act as potent antagonists. Here, we describe the characterization of compounds AMG0635, AMG2850, AMG8788, AMG9678, and Compound 496. All compounds potently inhibited the menthol and cold-induced increase in intracellular calcium in cells expressing rat TRPM8 (Figure 1; Table 1). None of the compounds activated TRPM8 at concentrations up to 40 μM, as measured by an aequorin luminescence assay that measures an increase in intracellular calcium in cells expressing TRPM8, indicating that they do not act as partial agonists. The rank order of the compound potency as antagonists at rat TRPM8 activated by menthol is: Compound 496 > AMG9678 > AMG0635 > AMG8788 > AMG2850. All compounds appeared to be more potent at blocking cold activation of TRPM8 compared to blocking menthol activation (Table 1). All compounds were found to be selective for TRPM8 relative to the recombinant TRP family members that we have tested (allyl isothiocyanate activated TRPA1, capsaicin activated TRPV1, 2-Aminoethoxydiphenyl borate activated TRPV3, and 4α-phorbol 12, 13-didecanoate activated TRPV4 (Table 1). The plasma half-life (T_1/2_) of the antagonists in rats for AMG0635, AMG2850, AMG8788, AMG9678, and Compound 496 is 2.8, 3.5, 6.7, 7.6, and 3.4 h, respectively.

**Table 1:** **IC**_**50**_values of TRPM8 antagonists at different TRP channels activated by specific agonists. Values shown are in nanomolar except where indicated with * are shown in μM. NA = not available

**Antagonist**	**TRPM8 Menthol (Cold)**	**TRPA1 (AITC)**	**TRPV1 (Capsaicin)**	**TRPV3 2-APB**	**TRPV4 (4αPDD)**
AMG8788	63.2 ± 31.7 (16 ± 14)	1 ± 0.7*	>20*	>20*	>20*
AMG0635	57.2 ± 0.1 (5.5 ± 3.4)	4.5 ± 1.6*	>20*	>20*	>20*
AMG9678	31.2 ± 8.3 (6.2 ± 1.9)	0.6 ± 0.4*	>20*	>20*	>20*
Compound 496	25.8 ± 6.6 (12 ± 0.9)	5.6 ± 2.4*	4.3*	>10*	>10*
AMG2850	156 ± 110 (7.3 ± NA)	>20*	>10*	>10*	>10*

### TRPM8 blockade in vivo elicits a transient decrease in body temperature

**Figure 2:**
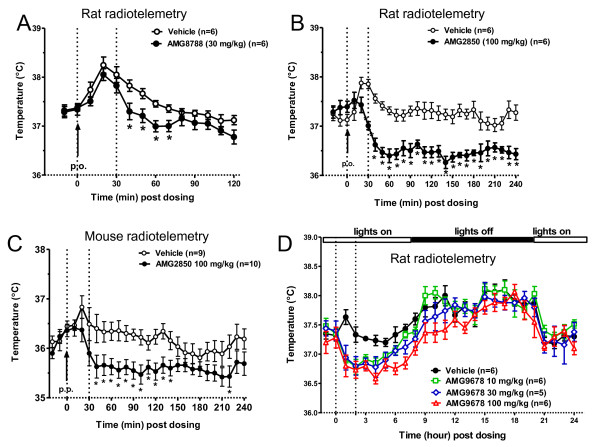
** Effects of TRPM8 antagonists on body temperature (T**_**b**_**) in rats or mice.** Data are presented as mean ± S.E.M. of temperature collected for every 10 min. Statistical significance is relative to the vehicle (one tail unpaired *t*-test). Baseline T_b_ was collected for 20–30 min before compound administration (p.o.) at time 0 and post dosing every 10 min for 120 min (**A**) or 240 min ( **B** &**C**). The stress-induced transient increase in T_b_ seen right after antagonist administration is indicated by vertical dotted lines. A) AMG8788 dosed at 30 mg/kg significantly decreased rat T_b_ by 0.53°C at 40 min (*t*_10_ = 2.55; *p* < 0.05). B) AMG2850 dosed at 100 mg/kg significantly decreased rat T_b_ by 0.98°C at 140 min (*t*_10_ = 4.38; *p* < 0.001). C) AMG2850 dosed at 100 mg/kg significantly decreased mouse T_b_ by 0.73°C at 100 min (*t*_17_ = 2.99; *p* < 0.001). **D**) TRPM8 antagonist AMG9678 induced decrease in body temperature is transient in nature. Statistical significance is relative to vehicle (one way ANOVA followed by Dunnett’s Multiple Comparison Test). Baseline T_b_ was collected at 30 min before compound administration (p.o.) at time 0 and post dosing every 1 h for 24 h. At 100 mg/kg, AMG9678 significantly decreased T_b_ by 0.83°C at 1 hour (*F*_3,22_ = 6.46, p < 0.01), whereas at the same time, the maximum decrease of T_b_ was 0.7 and 0.72 ^0^ C at 30 mg/kg and 10 mg/kg, respectively.

Since agonists of TRPM8, icilin and menthol are known to increase T_b_[32,33], we evaluated the effects of all five TRPM8 antagonists on T_b_ in rats or mice implanted with radiotelemetry probes. Different oral doses have been chosen based on the potency and pharmacokinetic properties of the antagonists. All antagonists lowered T_b_ with an overall maximum decrease of ~0.98°C (Table 2 and Figure 2). In a 2 h T_b_ recording experiment, AMG8788 at 30 mg/kg (p.o.) produced a significant decrease of T_b_ from 40 min (*t*_10_ = 2.55; *p* < 0.05) to 70 min ( *t*_10_ = 2.61; *p* < 0.05) (Figure 2A) post dosing. The maximum decrease in T_b_ was 0.53°C at 40 min and plasma concentration was 1.5 ± 0.6 μM at 2 h post dosing. In a 4 h T_b_ recording experiment, AMG2850 at 100 mg/kg (p.o.) produced a significant decrease of T_b_ from 40 min (*t*_10_ = 2.26; *p* < 0.05) to 4 h post-dosing ( *t*_10_ = 4.38; *p* < 0.001) (Figure 2B). The maximum decrease in T_b_ was 0.98°C at 140 min (*t*_10_ = 4.38; *p* < 0.001) post dosing and plasma concentration was 22 ± 0.8 μM at 4 h post dosing. In a 2 h T_b_ recording experiment, AMG0635 at 3 mg/kg (p.o.) produced a significant decrease of T_b_ from 40 min (*t*_10_ = 1.89; *p* < 0.05) to 120 min ( *t*_10_ = 5.88; *p* < 0.0001) post-dosing. The maximum decrease in T_b_ was 0.47°C at 120 min and plasma concentration was 0.38 ± 0.04 μM at 120 min post dosing (Table 2). In a 4 h T_b_ recording experiment, Compound 496 at 30 mg/kg (p.o., n = 6) produced a significant decrease of T_b_ from 30 min (*t*_10_ = 2.46; *p* < 0.05) to 180 min ( *t*_10_ = 2.64; *p* < 0.05) post dosing. The maximum decrease in T_b_ was 0.64°C at 100 min (*t*_10_ = 3.24; *p* < 0.01) and plasma concentration was 14.9 ± 0.95 μM at 100 min post dosing (Table 2).

**Table 2:** **Effect of different TRPM8 antagonists on T**_**b**_**in rats. P value is for comparing compound administered rat T**_**b**_**with vehicle administered rat T**_**b**_**. End of the study plasma concentration is reported in μM. Asterisk indicates one-way ANOVA followed by Dunnett's MCT**

**Compound**	**Dose mg/kg (route)**	**Max T**_**b**_**decrease (°C)**	*P*value *	**Time post dosing (min)**	**Plasma concentration**
AMG0635	3 (p.o.)	0.47	p < 0.05	120	0.38 ± 0.04
AMG8788	30 (p.o.)	0.53	p < 0.05	40	1.5 ± 0.6
AMG9678	10 (p.o.)	0.72	p < 0.001	60	0.04 ± 0.006
AMG9678	30 (p.o.)	0.70	p < 0.01	60	0.34 ± 0.1
AMG9678	100 (p.o.)	0.83	P < 0.05	60	0.36 ± 0.12
AMG2850	100 (p.o.)	0.98	p < 0.0001	140	22 ± 0.8
Compound 496	30 (p.o.)	0.64	p < 0.01	100	14.9 ± 0.95

Further, AMG2850 was also tested in mice at 100 mg/kg in a 4 h study. There was a significant decrease of T_b_ from 40 min (*t*_17_ = 2.11; *p* < 0.05) to 140 min ( *t*_17_ = 2.31; *p* < 0.05) with a maximum decrease of 0.73°C at 100 min ( *t*_17_ = 2.99; *p* < 0.01) and plasma concentration was 54 ± 5.6 μM at 4 h post dosing (Figure 2C).

To understand whether decrease in T_b_ correlates with plasma concentrations of TRPM8 antagonists, we administered different oral doses of AMG9678 to rats and monitored their temperatures for 24 h (Figure 2D). In this study, AMG9678 produced a significant and somewhat dose-dependent decrease in T_b_ at 10, 30 and 100 mg/kg (p.o.). The greatest decrease of T_b_ relative to vehicle group was 0.83°C at 1 h post dosing in 100 mg/kg administered rats, whereas 0.70°C and 0.72°C decrease in T_b_ was observed at 30 and 10 mg/kg, respectively (*F*_3,22_ = 6.46, p < 0.01) At 100 mg/kg, significant decrease in T_b_ was observed from 1 to 8 h (*F*_3,22_ = 3.99, p < 0.05). At 30 mg/kg dose, decrease in T_b_ lasted for 4 h (*F*_3,22_ = 6.35, p < 0.01), whereas at 10 mg/kg, this effect lasted for only 3 h (*F*_3,22_ = 8.56, p < 0.001). The plasma concentrations at the end of the study (24 h post dosing) were: 355.8 ± 116.4 nM at 100 mg/kg, 342.6 ± 97.6 nM at 30 mg/kg, and, 42.2 ± 6.4 nM at 10 mg/kg, respectively.

### The magnitude of TRPM8 blockade-induced decrease in body temperature is reduced after repeated dosing of an antagonist

**Figure 3:**
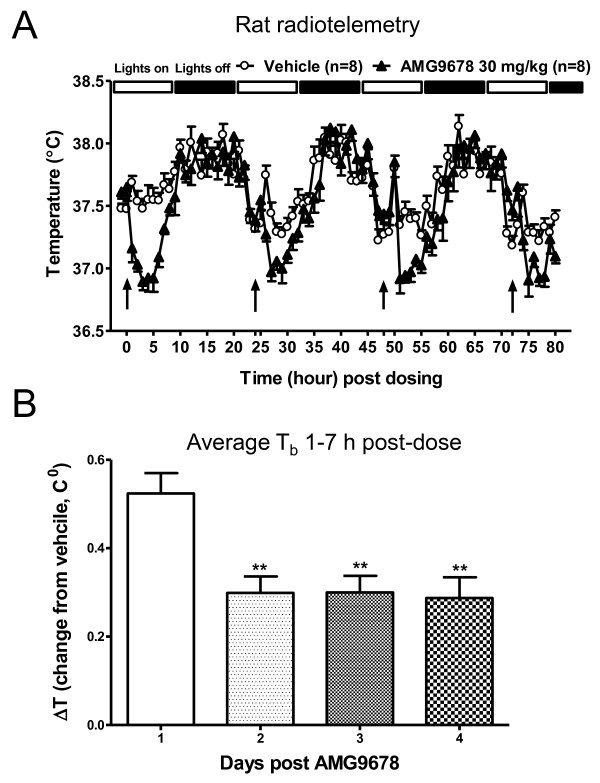
** AMG9678-induced decrease in T**_**b**_**partially attenuates after repeat dosing in rats.** AMG9678 was administered (p.o.) each day at 9:00 am for 4 days as indicated by an arrow. T_b_ data was collected every hour for 80 h and are presented as mean ± S.E.M. **A**) AMG9678 at 30 mg/kg produced a significant decrease of T_b_ by 0.62°C at 5 h (*t*_14_ = 4.27, *p* < 0.001), 0.47°C at 26 h ( *t*_14_ = 4.95, *p* < 0.001), 0.51°C at 52 h ( *t*_14_ = 5.01, *p* < 0.0001), and 0.38°C at 75 h ( *t*_14_ = 2.68, *p* < 0.01), respectively. The decrease in T_b_ lasted for 7 h post 1^st^ dosing, 5 h post 2^nd^ dosing, 5 h post 3^rd^ dosing and 6 h post 4^th^ dosing, respectively. **B**. AMG9678-induced decrease in T_b_ reduced on day 2–4 compared to day 1. Bars represent mean ± S.E.M. of ΔT of average 1–7 h post dosing on days 1–4.

When administered as a single dose, AMG9678-induced decrease in T_b_ was transient in nature, with a peak effect occurring within 1 h post dosing and sustained up to 12 h. To evaluate the effect of repeated dosing on TRPM8 antagonist-induced decrease in T_b_, we administered AMG9678 once daily for 4 consecutive days to rats and recorded T_b_ for 80 h (Figure 3A). AMG9678 at 30 mg/kg produced a significant effect with maximum T_b_ decrease of 0.62°C at 5 h (*t*_14_ = 4.27, *p* = 0.001), 0.47°C at 26 h ( *t*_14_ = 4.95, *p* < 0.001), 0.51°C at 52 h ( *t*_14_ = 5.01, *p* < 0.0001), and 0.38°C at 75 h ( *t*_14_ = 2.68, *p* < 0.01), respectively, indicating a reduction of T_b_ decrease after repeated dosing. The decrease in T_b_ lasted for 7 h after the first dosing, 5 h post second dosing, 5 h post third dosing and 6 h post fourth dosing.

The average change in temperature on each day (1–7 hours post dosing) of individual animals in the drug group relative to the average temperature of the vehicle group is presented in Figure 3B. AMG9678-induced 0.52°C decrease in T_b_ relative to vehicle on the 1^st^ day, and 0.30°C, 0.30°C, and 0.29°C on the 2^nd^, 3^rd^ and 4^th^ day, respectively. One-way ANOVA followed by Tukey’s multiple comparisons *post hoc* test indicates that the decrease in T_b_ on day 1 is a significantly different from each of the subsequent three days (p < 0.001) and that the decrease in T_b_ on days 2–4 are not significantly different from each other. Even though the decrease in T_b_ on day 4 is still significant compared to the vehicle, the fact that the decrease in T_b_ on days 2–4 is significantly less than that on day 1 suggests that there may be an attenuation following repeated dosing. The plasma concentration at the end of study (80 h post first dosing, 7 h post fourth dosing) was 0.41 ± 0.03 μM.

## Discussion

### TRPM8 channels involved in body temperature maintenance under cold conditions

Menthol and icilin activate TRPM8 and are known to cause an increase in T_b_[32,33,38-40], however, it is not known if TRPM8 itself is involved in T_b_ maintenance. To evaluate whether TRPM8 channels are involved in T_b_ maintenance, we have characterized five distinct compounds as potent and selective antagonists of TRPM8 and studied their effects on T_b_ in rats and mice. Surprisingly, all compounds induced a small but statistically significant decrease in T_b_. We believe that the decrease in T_b_ is the result of TRPM8 blockade *in vivo* because the antagonists used in our studies are selective for TRPM8 compared to the other TRP channels that we tested. Some of the antagonists used in this study showed weak antagonism at TRPA1 (16 to 80-fold less potent compared to TRPM8 antagonism), however, TRPA1 antagonism *in vivo* with A-967079, a potent and selective antagonist did not alter T_b_[41], which suggests that TRPM8 antagonism is responsible for decrease in T_b_ in the current studies. While this manuscript was in preparation, a structurally different TRPM8 selective antagonist, 1-phenylethyl-4-(benzyloxy)-3-methoxybenzyl(a-aminoethyl)carbamate also reported to cause a decrease in T_b_ in wild type but not in TRPM8 knockout mice suggesting that the decrease in T_b_ is exclusively mediated by TRPM8 [42]. More recently, we reported that another structurally different TRPM8 selective antagonist, M8-B elicits a decrease in T_b_ only when ambient temperatures reach to the activation threshold of TRPM8 in rats (and mice) but did not affect T_b_ in TRPM8 knockout mice [43]. The mechanisms of TRPM8 antagonist-induced decrease in T_b_ include: i) transient delay in onset of the tail-skin vasoconstrictor response to cold environment, ii) transient decrease in oxygen consumption (metabolic heat production), and iii) transient decrease in brown fat thermogenesis [43]. Based on the results reported here, studies by Knowlton et al. [42], and Almeida et al. [43], we conclude that TRPM8 is involved in T_b_ maintenance under cold ambient temperatures. Since all the radiotelemetry experiments reported here are done at an ambient temperature of 20 ± 2°C, a temperature range that activates TRPM8 and plays a role in thermoregulation, we suggest that TRPM8 appears to be not tonically active but plays a role in T_b_ maintenance only in cold environment.

### Members of ThermoTRP channels act as counterbalancing thermosensors for the T_b_ maintenance

Antagonists of TRPV1 alone causing T_b_ modulation revealed that these channels are tonically active. Since TRPA1, TRPM8, TRPC5, TRPV3, TRPV4, and TRPV1 cover the typical environmental cold and heat sensing range to act as thermosensors [14,18-20], activation of these channels perhaps triggers behavioral (heat or cold seeking behavior) as well as autonomic thermoeffectors (vasomotor tone and thermogenesis) to maintain the T_b_ at 37°C (thus constitute a basis for T_b_ homeostasis). It is possible that some of the thermoTRP channels may be tonically active (TRPV1 and other channels with activation thresholds close to T_b_) whereas others may only be active when ambient temperatures reach their activation thresholds (TRPM8 and perhaps other low temperature activated channels).

Tonically active TRPV1 channels are reported to be present in the visceral nerve terminals [35] but it is not clear where other tonically active channels are located. Independent of their location, tonically active ‘thermosensor’ channels (TRPV1 and perhaps others) may work as counterbalancing thermoregulators simply by their level of activation (could be measured as maximum open probability *P*_o_). A change in *P*_o_ of a thermosensor channel alters T_b_ through recruitment of some or all thermoeffector loops and in turn altered T_b_ itself might trigger a change in *P*_o_ of a counterbalancing thermosensor(s), which will then engage some or all thermoeffector loops in the opposite direction to bring T_b_ back toward 37°C. This perhaps constitutes a fundamental basis for T_b_ homeostasis. It is demonstrated clearly that modulation of thermosensors (e.g., TRPM8 and TRPV1 by agonists and/or antagonists) engages thermoeffectors to alter T_b_[34,35,39,40], however the demonstration of altered T_b_ itself changing the *P*_o_ (activating) of another thermosensor awaits.

### Does ThermoTRP role in T_b_ regulation pose a road block to develop antagonists as therapeutics?

It is reported that TRPV1 antagonists, AMG 517, AZD 1386 and MK-2295 raised T_b_ in humans and all three of them appear to be no longer in clinical development. AMG 517 is dropped out of clinical development due to hyperthermia [44], MK-2295 due to rightward shift in heat tolerance (risk of accidental heat injuries), and AZD 1386 for lack of efficacy in Phase II trials [36].

Since TRPM8 antagonists elicit only a small and transient decrease in T_b_, and only under ambient temperatures that activate TRPM8 channels in the skin nerve terminals ([43], this study), the decrease in T_b_ appears to show attenuation after repeated dosing of an antagonist (this study), and it is known that many pharmaceutical and neutraceutical compounds cause a 1–2°C decrease in T_b_[45], effects on thermoregulation might not pose an issue to develop TRPM8 antagonists as therapeutics.

### Concluding remarks

We propose that thermoTRP channels play both physiological (thermoregulation by acting as thermosensors) and pathophysiological (hyperalgesia) roles. Among the ones involved in thermoregulation, some (e.g., TRPV1) mediate thermoeffectors exclusively [35] whereas others (e.g., TRPM8 [43] and other thermoTRPs) engage both behavioral [40,43] as well as autonomic thermoeffectors [43]. It is known that TRPC5 is activated by cold [46] and TRPV3, and TRPV4 are activated by warm temperature [15,47], but it is not known if blockade of these channels modulates T_b_. However, based on the fact that TRPM8 and TRPV1 antagonists affect T_b_, it is plausible that some of the other thermoTRP channels may also be involved in T_b_ homeostasis. Future studies should reveal the role of additional TRP channels in thermoregulation.

## Methods

### Luminescence readout assay for measuring intracellular calcium

Stable CHO cell lines expressing TRPA1, TRPM8, TRPV1, TRPV3, and TRPV4 were generated using tetracycline inducible T-REx^TM^ expression system from Invitrogen, Inc (Carlsbad, CA). In order to enable a luminescence readout based on intracellular increase in calcium [48], each cell line was also co-transfected with pcDNA3.1 plasmid containing jellyfish aequorin cDNA. Twenty four hours before the assay, cells were seeded in 96-well plates and TRP channel expression was induced with 0.5 μg/ml tetracycline. On the day of the assay, culture media was removed and cells were incubated with assay buffer (F12 containing 30 mM HEPES for TRPA1, TRPM8, and TRPV3; F12 containing 30 mM HEPES, 1 mM CaCl_2_, and 0.3% BSA for TRPV4) containing 15 μM coelenterazine (P.J.K, Germany) for 2 h. Antagonists were added for 2.5 min prior to addition of an agonist except for cold activation of TRPM8 (1 min prior to addition of cold buffer ≤10°C). Luminescence was measured by a CCD camera based FLASH-luminometer built by Amgen, Inc. The following agonists were used to activate TRP channels: 80 μM allyl isothiocyanate for TRPA1, 100 μM menthol for TRPM8, 0.5 μM capsaicin for TRPV1, 200 μM 2-Aminoethoxydiphenyl borate for TRPV3, and 1 μM 4α-phorbol 12,13-didecanoate for TRPV4 [49]. Compound activity was calculated using either ActivityBase or GraphPad Prism 4.01 (GraphPad Software Inc, San Diego, CA).

### Pharmacokinetics

For T_1/2_ determination, intravenous dosing of each compound in DMSO was performed via the jugular vein in male Sprague Dawley rats (n = 3 animals per study). At designated time points, blood was collected via the femoral artery in rat. Blood was collected and processed for plasma by centrifugation. For exposure measurements in radiotelemetry experiments, at the end of T_b_ recording blood was collected from the animals via cardiac puncture and processed for plasma by centrifugation. Plasma was then transferred into a 96-well container and stored in a freezer maintained at approximately −70°C. Plasma concentrations of each test article were measured using sensitive LC/MS/MS methods optimized for each compound. Non-compartmental pharmacokinetics analysis of plasma concentrations was conducted using WinNonlin Enterprise v.5.1.1 (Pharsight Corporation, Mountain View, CA).

### Radiotelemetry in naïve rats

#### Animals

Male Sprague Dawley rats (Harlan Laboratories, Indianapolis, IN) weighing 200–350 g (6–12 weeks of age) and male C57BL/6 mice (Taconic, Hudson, NY) weighing 24–38 g (10–15 weeks of age) were single-housed and acclimated for 1-week in the animal care facility prior to start of experiments. The temperature in the room used for animal holding and radiotelemetry experiments was maintained at 20 ± 2°C.

#### Radiotelemetry probe implantation

To implant the radiotelemetry probe (model ER-4000 PDT; Mini Mitter, Bend, OR), rats or mice were anesthetized using isoflourane (IsoFlo, Abbott Laboratories, Chicago, IL) at a concentration of 4% isoflourane at 4 L/min oxygen flow. While animals rested in a supine position, fur of the mediolateral abdominal area was clipped and skin was cleaned with Betadine Solution (Purdue Frederick Company, Stamford, CT) followed by 70% alcohol in water. A 1 cm incision was made through the skin and abdominal wall, such that a sterilized probe could be inserted into the peritoneal cavity. Once inserted, the surgical site was closed with 5–0 monocryl suture material (Ethicon Inc, Somerville, NJ). Animals were returned to a clean home-cage for 2 days of recovery prior to experiments.

#### Body temperature (T_b_) measurement

Overnight acclimation to the testing room occurred prior to the experiment by placing home cages of probe-implanted, single-housed animals on the radiotelemetry receivers. During a less than 24 h experiment, T_b_ was recorded every 10 min starting with baseline T_b_ (prior to drug administration) for up to 30 min and then post dosing for 2–4 h. For a 24 h or longer study, T_b_ was recorded every hour for 2 h (baseline) and then post dosing up to 80 h. Animals (5–10 per group) were administered either vehicle (5% Tween-80/Ora-plus) or a single to multiple doses (dose–response study) of TRPM8 antagonists, or once daily dosing of an antagonist for 4 days, in a dose volume of 5 ml/kg (in vehicle, oral gavage). Blood samples were collected at the end of the T_b_ recording for pharmacokinetic analysis.

#### Statistical analysis

All T_b_ data are presented as mean ± S.E.M. In the single dose study, statistical significance of drug treated groups was determined by comparison to the vehicle treated group using multiple, independent one-tailed, unpaired *t*-tests at each time point post-drug administration (Table 2; Figure 2A-C). In the dose–response study, all T_b_ data were compared to the vehicle control group using multiple, independent one-way analysis of variance (ANOVA) tests followed by Dunnett’s multiple comparisons post-hoc test for significance at each time point (AMG9678 data in Table 2; Figure 2D). In order to assess whether the effect on temperature may change following repeated dosing, a one-way ANOVA followed by Tukey’s multiple comparisons post hoc test was conducted to compare this change in temperature relative to vehicle for each of the 4 days (Figure 3B).

## Abbreviations

T_b_: Deep body temperature; PIP_2_: Phosphatidylinositol 4,5-bisphosphate; AMG0635: (R)-N-(pyridin-3-yl)-1-(4-(trifluoromethyl)phenyl)-3,4-dihydroisoquinoline-2(1 H)-carboxamide; AMG2850: (R)-8-(4-(trifluoromethyl)phenyl)-N-((S)-1,1,1-trifluoropropan-2-yl)-5,6-dihydro-1,7-naphthyridine-7(8 H)-carboxamide; AMG8788: (R)-N-(4-fluorophenyl)-1-(4-(trifluoromethyl)phenyl)-3,4-dihydroisoquinoline-2(1 H)-carboxamide; AMG9678: (R)-1-(4-(trifluoromethyl)phenyl)-N-((S)-1,1,1-trifluoropropan-2-yl)-3,4-dihydroisoquinoline-2(1 H)-carboxamide; Compound 496: 4-(N-(3-methylbenzo[b]thiophen-2-yl)-N-(4-(trifluoromethoxy)benzyl)sulfamoyl)benzoic acid.

## Competing interests

All authors are employed by a for profit company, Amgen Inc.
